# Emotional Stability and Emotional Eating in Medical Students: Exploring Indirect Associations Involving Anxiety and Depressive Symptoms

**DOI:** 10.3390/nu18152499

**Published:** 2026-08-03

**Authors:** Lorena Mihaela Grebenisan, Bianca Larisa Abalasei, Andreea Sima-Comaniciu, Aurel Nireștean, Elena Gabriela Strete, Alex Claudiu Boaca, Alexandru-Andrei Ujlaki-Nagi, Dora Mihaela Cimpean, Rareș Manolachi, Corina Mărginean, Emese Lukacs

**Affiliations:** 1 Department of Psychiatry, George Emil Palade University of Medicine, Pharmacy, Science and Technology of Targu Mures, 540142 Targu Mures, Romania; lorena-mihaela.muntean@umfst.ro (L.M.G.); aurel.nirestean@umfst.ro (A.N.); elena.buicu@umfst.ro (E.G.S.); alex.boaca@umfst.ro (A.C.B.); alexandru.ujlaki@umfst.ro (A.-A.U.-N.); emese.lukacs@umfst.ro (E.L.); 2 Doctoral School, George Emil Palade University of Medicine, Pharmacy, Science and Technology of Targu Mures, 540142 Targu Mures, Romania; andreea.comaniciu@umfst.ro; 3 Department of Ethics and Social Sciences, George Emil Palade University of Medicine, Pharmacy, Science and Technology of Targu Mures, 540142 Targu Mures, Romania; dora.cimpean@umfst.ro; 4 Faculty of Medicine, “Iuliu Hațieganu” University of Medicine and Pharmacy, 400012 Cluj-Napoca, Romania; manolachirares65@gmail.com; 5 Department of Oncology and Palliative Care, George Emil Palade University of Medicine, Pharmacy, Science and Technology of Targu Mures, 540142 Targu Mures, Romania; corina.marginean@umfst.ro

**Keywords:** anxiety symptoms, depression symptoms, emotional stability, emotional eating

## Abstract

Background/Objectives: Emotional stability is a personality trait associated with emotion regulation and adaptation to stress. Low emotional stability has been associated with anxiety, depressive symptoms, and emotional eating, but the mechanisms underlying these relationships among medical students remain unclear. This study examined the association between emotional stability and emotional eating and explored indirect statistical associations involving anxiety and depressive symptoms using exploratory parallel and serial path analyses. Methods: A cross-sectional observational study was conducted among 291 medical students. Emotional stability was assessed using the DECAS Personality Inventory, emotional eating using the DEBQ (Dutch Eating Behavior Questionnaire), anxiety symptoms using the GAD-7 (Generalized Anxiety Disorder-7), and depressive symptoms using the PHQ-9 (Patient Health Questionnaire-9). Spearman correlations and exploratory parallel and serial path analyses were performed using the PROCESS macro for SPSS Statistical Calculator v26 with 5000 bootstrap samples. Results: Emotional stability was negatively correlated with anxiety (ρ = −0.546; *p* < 0.001), depressive symptoms (ρ = −0.603; *p* < 0.001), and emotional eating (ρ = −0.383; *p* < 0.001). Anxiety (ρ = 0.380; *p* < 0.001) and depressive symptoms (ρ = 0.454; *p* < 0.001) were positively correlated with emotional eating. Only depressive symptoms showed a significant indirect statistical association linking emotional stability and emotional eating (B = −0.0139; 95% bootstrap CI: −0.0223 to −0.0068). In the serial path analysis, only the model specifying anxiety as the first intermediate variable showed a significant indirect effect (B = −0.0065; 95% bootstrap CI: −0.0109 to −0.0032). Conclusions: Depressive symptoms showed the strongest indirect association linking emotional stability and emotional eating in medical students. These findings should be interpreted as being consistent with the proposed statistical model and do not establish temporal or causal relationships.

## 1. Introduction

In today’s society, anxiety and depressive symptoms are highly prevalent in the general population and represent major public health concerns because of their detrimental impact on quality of life, highlighting the importance of identifying psychological risk factors across different populations [1,2,3,4]. Medical students are exposed throughout their university education to specific stressors, including high information volume, frequent assessments, academic competition, and progressive contact with the clinical environment [5,6]. These challenges can influence mental health and lifestyle behaviors, including eating behavior [7,8]. 

Eating behavior is influenced not only by physiological hunger but also by psychological and emotional factors. Both anxiety and depressive symptoms are associated with emotional eating or other maladaptive eating behaviors [9,10,11]. In this context, eating behavior can function as an emotion regulation mechanism, with individuals resorting to food consumption to cope with negative emotions such as stress, anxiety, or sadness [12]. From a nutritional perspective, emotional eating represents an important eating behavior because it has been consistently associated with increased consumption of energy-dense and highly palatable foods, poorer diet quality, excess energy intake, weight gain, overweight and obesity, as well as adverse metabolic outcomes [13,14,15]. Accordingly, current healthcare approaches increasingly recognize the importance of integrating psychological, nutritional, and metabolic perspectives in the management of obesity and related metabolic conditions [16].

Emotion regulation refers to the ability to monitor and modify emotional responses according to situational demands [17]. Existing evidence suggests that emotional regulation difficulties are associated with both increased levels of anxiety and depression and maladaptive eating behaviors [18,19]. Furthermore, emotion regulation has been recognized as a transdiagnostic process across eating pathology, highlighting its central role in maladaptive eating behaviors, including emotional eating [20]. Recent mediation studies further support the role of emotion regulation in explaining indirect relationships between individual characteristics and psychological outcomes, suggesting that emotion regulation difficulties may contribute to psychological distress through underlying psychological processes [21]. Within this framework, emotional eating can be understood as a maladaptive strategy for coping with negative emotions, suggesting that anxiety and depressive symptoms may represent plausible psychological pathways linking emotional stability and emotional eating [22,23].

Personality traits influence how individuals perceive stress, regulate emotions, and adopt health-related behaviors and are associated with eating behavior as well as vulnerability to anxiety and depressive symptoms [24,25,26]. Growing evidence suggests that personality traits play an important role in the psychological functioning of medical students, influencing emotional adjustment, interpersonal functioning, and health-related behaviors [27]. Among the Big Five personality dimensions, emotional stability appears to play a particularly important role. Individuals with lower emotional stability tend to experience negative emotions more frequently, perceive stressful situations as more threatening, and rely on less effective emotional regulation strategies [28,29]. Consequently, lower emotional stability has consistently been associated with higher levels of anxiety and depressive symptoms [30,31], which may increase vulnerability to maladaptive eating behaviors, particularly emotional eating [32].

Understanding the role of anxiety and depressive symptoms in the association between personality traits and eating behavior is important for identifying individuals at increased risk of developing maladaptive eating behaviors and for developing preventive interventions targeting mental health and lifestyle [33,34].

Despite evidence supporting the existence of associations between emotional stability, anxiety and depressive symptomatology, and eating behavior, the relationships among these variables remain insufficiently understood [25,35]. In particular, it remains unclear whether anxiety and depressive symptoms are involved in the indirect statistical associations between emotional stability and emotional eating among medical students, a population exposed to high levels of psychological stress. A better understanding of these relationships may help identify students at increased risk of maladaptive eating behaviors and inform preventive strategies to promote mental health and healthier eating behaviors [36,37].

Considering these aspects and the fact that medical students represent a particularly relevant population given the unique academic and psychological demands, the present study aimed to investigate the relationship between emotional stability and emotional eating and to explore the indirect statistical associations involving anxiety and depressive symptoms using parallel and serial path analyses. Based on the existing literature, we formulated the following hypotheses:

**H1.**  *Emotional stability is negatively correlated with anxiety and depressive symptoms.*

**H2.**  *Anxiety and depressive symptoms are positively correlated with emotional eating.*

**H3.**  *Emotional stability is negatively correlated with emotional eating.*

**H4.**  *Anxiety and depressive symptoms are expected to be involved in significant indirect statistical associations linking emotional stability and emotional eating.*

## 2. Materials and Methods

### 2.1. Study Design and Recruitment

A cross-sectional, monocentric observational study was conducted between 1 May and 1 June 2026 among medical students from the first to the sixth year of study at the “George Emil Palade” University of Medicine, Pharmacy, Science, and Technology of Târgu Mureș. Participation in the study was voluntary and anonymous, and data were collected electronically. Before completing the questionnaires, participants were informed about the study objectives and provided informed consent. The survey was administered using Google Forms, and the survey link was distributed through the official social media groups corresponding to each year of study. The study invitation specified that only medical students enrolled at the university were eligible to participate.

### 2.2. Sample Size Estimation

An a priori power analysis was conducted using G*Power version 3.1.9.7, assuming a medium effect size (f^2^ = 0.15), α = 0.05, and a statistical power of 0.80. The minimum required sample sizes were 82 participants for the correlation analyses and 77 participants for the regression analyses. Post hoc power and sensitivity analyses confirmed that the final sample of 291 participants provided excellent statistical power for all analyses.

### 2.3. Participants

Of the 294 students who completed the survey, 291 met the eligibility criteria and were included in the final analyses. Of the total participants, 77.32% were women and 22.68% were men, with a mean age of 23.34 ± 2.06 years.

Inclusion criteria were enrollment in the Medicine study program within the Faculty of Medicine, a minimum age of 18 years, and agreement to participate by accepting informed consent. Participants who did not complete all questionnaires or whose responses were incomplete and obtained high scores on the internal validation scales of the DECAS Personality Inventory were excluded from the final analysis.

### 2.4. Assessment of Study Variables

#### 2.4.1. Emotional Stability

Emotional stability was assessed using the DECAS Personality Inventory, a psychometric instrument based on the Big Five personality model. The instrument was developed by Sava and collaborators. The instrument assesses five personality dimensions: openness, extraversion, conscientiousness, agreeableness, and emotional stability. It consists of 97 true/false items. In this study, the emotional stability dimension was used, which reflects the individual’s tendency to maintain emotional balance and manage stress and negative emotions effectively [38]. The inventory has been validated in the Romanian population and has demonstrated adequate psychometric properties. For the emotional stability subscale, the authors reported an internal consistency coefficient of α = 0.75. The scale has three internal validation scales to detect biased responses [38].

#### 2.4.2. Eating Behavior

Eating behavior was assessed using the Dutch Eating Behavior Questionnaire (DEBQ), an instrument developed by Van Strien and colleagues to assess individual eating behavior styles. The questionnaire comprises three subscales: emotional eating, eating driven by external factors, and restrictive eating [39]. The emotional eating subscale assesses the tendency to use food to manage negative emotions and affective states. People with high scores on this dimension tend to consume food more frequently in situations characterized by stress, anxiety, sadness, loneliness, or other unpleasant emotional experiences [39]. The questionnaire has been validated in the Romanian population and has demonstrated adequate psychometric properties, with a Cronbach’s α coefficient of 0.95 for the emotional eating subscale [40]. Only the emotional eating subscale was included in the present analyses because the primary objective of the study was to investigate the associations between emotional stability, anxiety, depressive symptoms, and emotional eating. The external eating and restrained eating subscales assess different dimensions of eating behavior that were beyond the scope of the present investigation [39].

#### 2.4.3. Anxiety Symptoms

Anxiety symptoms were assessed using the Generalized Anxiety Disorder-7 (GAD-7), a self-report questionnaire developed by Spitzer, Kroenke, Williams, and Löwe to rapidly assess the severity of anxiety symptoms. The instrument consists of seven items rated on a four-point Likert scale ranging from 0 (“not at all”) to 3 (“nearly every day”), yielding a total score between 0 and 21. Higher scores indicate greater symptom severity, with scores of 0–4 indicating minimal anxiety, 5–9 mild anxiety, 10–14 moderate anxiety, and 15–21 severe anxiety [41]. The Romanian version of the GAD-7 has demonstrated adequate psychometric properties, with Cronbach’s alpha coefficients of 0.92 in a clinical sample and 0.75 in the general population [42].

#### 2.4.4. Depressive Symptoms

Depressive symptoms were assessed using the Patient Health Questionnaire-9 (PHQ-9), a nine-item self-report questionnaire designed to assess the severity of depressive symptoms experienced over the previous 2 weeks. The questionnaire was developed by Kroenke, Spitzer, and Williams. Items are rated on a four-point Likert scale ranging from 0 (“not at all”) to 3 (“nearly every day”), resulting in a total score ranging from 0 to 27, with higher scores indicating greater depression severity [43]. The Romanian version of the PHQ-9 demonstrated good psychometric properties in a study by Duica et al., with a Cronbach’s alpha coefficient of 0.88 among healthcare students [44].

## 3. Statistical Strategy

The study database was created and organized using Microsoft Excel (Microsoft Office Professional Plus 2021) before being imported into IBM SPSS Statistics for statistical analysis. Regression-based path analyses were conducted using the PROCESS macro for IBM SPSS Statistical Calculator v26 developed by Andrew F. Hayes. Initially, data were screened for missing values, incomplete responses, and response validity based on the internal validation scales of the DECAS Personality Inventory. Three participants were excluded from the initial sample: two due to extreme emotional stability scores exceeding three standard deviations from the mean, and one additional multivariate outlier identified using Mahalanobis distance. The final analytical sample consisted of 291 participants.

Descriptive statistics were calculated for all study variables. Categorical variables were reported as frequencies and percentages, whereas continuous variables were summarized using means and standard deviations, as well as medians and interquartile ranges. The normality of the continuous variables was assessed using the Shapiro–Wilk and Kolmogorov–Smirnov tests, with Lilliefors correction, together with visual inspection of histograms and P–P plots. Because most variables deviated from normality, Spearman’s rank correlation coefficients were used to examine bivariate associations among emotional stability, anxiety symptoms, depressive symptoms, and emotional eating.

Before conducting the regression-based path analyses, the assumptions underlying the regression models were evaluated. Residual normality was assessed using the Shapiro–Wilk and Kolmogorov–Smirnov tests, histograms, and standardized residual P–P plots. Homoscedasticity was evaluated through visual inspection of scatterplots of standardized residuals against standardized predicted values and formally tested using the Breusch–Pagan test. Multicollinearity was assessed using tolerance values and variance inflation factors (VIF). VIF values for the main predictors ranged from 1.02 to 1.61, indicating no evidence of problematic multicollinearity. Slightly higher VIF values (approximately 3.35) were observed for age and year of study, indicating no evidence of problematic multicollinearity and no substantial violations of the regression assumptions were identified.

Regression-based path analyses were conducted using the PROCESS macro for SPSS. A parallel path model was tested using Model 4, with emotional stability as the independent variable, emotional eating as the dependent variable, and anxiety and depressive symptoms entered simultaneously in the proposed path model. Because the previous literature describes a close and potentially bidirectional relationship between anxiety and depressive symptoms, without establishing a consistent temporal sequence [45,46], two alternative serial path models were subsequently examined using Model 6. This approach allowed the evaluation of two theoretically plausible path models while acknowledging that, given the cross-sectional design, neither model can establish the actual temporal ordering of these variables. In one model, anxiety symptoms were specified before depressive symptoms in the statistical model, whereas in the other model, depressive symptoms were specified before anxiety symptoms.

All regression-based path models were adjusted for sex, age, year of study and BMI. Indirect effects were estimated using 5000 bootstrap resamples. An indirect effect was considered statistically significant when the 95% bootstrap confidence interval did not include zero. Regression coefficients are reported as unstandardized coefficients (B), and statistical significance was set at *p* < 0.05.

## 4. Results

The study sample consisted of 291 medical students, most of whom were female (77.32%) and lived in urban areas (75.26%). Participants had a mean age of 23.34 ± 2.06 years. Nearly half of the students were in their sixth year of study (45.02%), followed by fifth-year students (17.87%). Regarding living arrangements, almost half of participants (49.83%) lived in rented accommodation, 25.43% in dormitories, 15.81% with their families, and 8.93% alone. Detailed characteristics of the study sample are presented in Table 1.

Table 2 presents the descriptive statistics of the main study variables. The mean emotional stability score was 47.88. The mean anxiety and depression scores were 11.10 and 11.03, respectively, while the mean emotional eating score was 2.41.

As shown in Table 3, emotional stability was strongly and negatively correlated with anxiety (ρ = −0.546, *p* < 0.001) and depression (ρ = −0.603, *p* < 0.001), and moderately negatively correlated with emotional eating (ρ = −0.383, *p* < 0.001). Anxiety was strongly positively correlated with depression (ρ = 0.668, *p* < 0.001) and moderately positively correlated with emotional eating (ρ = 0.380, *p* < 0.001). Depression was also moderately positively correlated with emotional eating (ρ = 0.454, *p* < 0.001).

**Figure 1:**
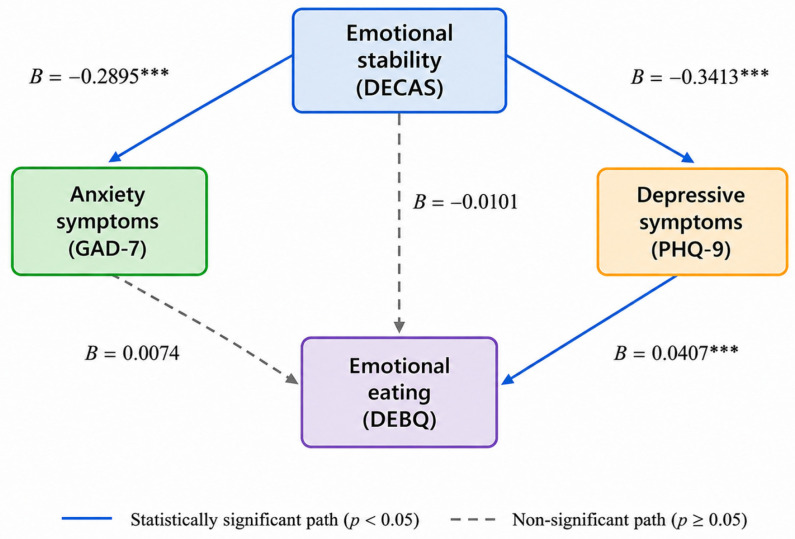
The parallel path model (PROCESS Model 4). The values on the arrows represent unstandardized regression coefficients (B). The solid arrows indicate statistically significant paths (*p* < 0.05), whereas the dashed arrows indicate non-significant paths. DECAS = DECAS Personality Inventory; PHQ-9 = Patient Health Questionnaire-9; GAD-7 = Generalized Anxiety Disorder-7; DEBQ = Dutch Eating Behavior Questionnaire. *** *p* < 0.001.

The results of the parallel path analysis are presented in Table 4 and Table 5, Figure 1. Emotional stability was significantly associated with lower levels of depressive symptoms (B = −0.3413, *p* < 0.001) and anxiety (B = −0.2895, *p* < 0.001). Depressive symptoms were significantly associated with emotional eating (B = 0.0407, *p* < 0.001), whereas anxiety symptoms were not significantly associated with emotional eating (B = 0.0074, *p* = 0.499). The total effect of emotional stability on emotional eating was significant (B = −0.0261, *p* < 0.001), whereas the direct effect became non-significant after accounting for anxiety and depressive symptoms in the path model (B = −0.0101, *p* = 0.0575). Bootstrap analyses based on 5000 resamples indicated a significant total indirect effect (B = −0.0160). Examination of the specific indirect effects showed a significant indirect effect of emotional stability on emotional eating through depressive symptoms (B = −0.0139), whereas the indirect effect of emotional stability on emotional eating through anxiety was not statistically significant (B = −0.0021).

**Figure 2:**
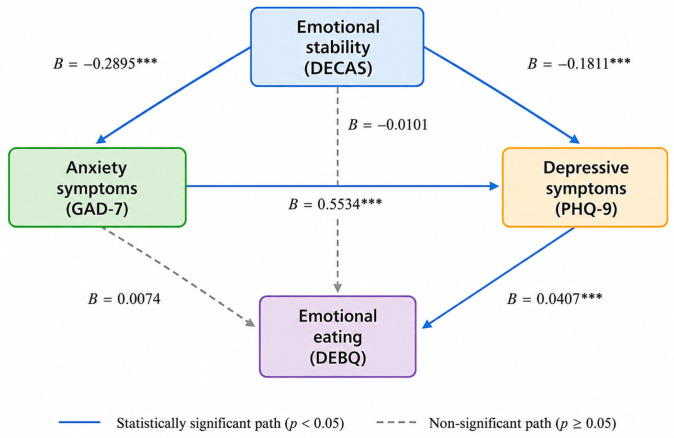
The serial path model with anxiety specified before depressive symptoms (PROCESS Model 6). The values on the arrows represent unstandardized regression coefficients (B). The solid arrows indicate statistically significant paths (*p* < 0.05), whereas the dashed arrows indicate non-significant paths. DECAS = DECAS Personality Inventory; PHQ-9 = Patient Health Questionnaire-9; GAD-7 = Generalized Anxiety Disorder-7; DEBQ = Dutch Eating Behavior Questionnaire. *** *p* < 0.001.

The results of the serial path analysis are presented in Table 6 and Table 7, Figure 2. Emotional stability was significantly associated with lower levels of anxiety (B = −0.2895, *p* < 0.001) and depressive symptoms (B = −0.1811, *p* < 0.001), while anxiety was significantly associated with depressive symptoms (B = 0.5534, *p* < 0.001). Depressive symptoms were significantly associated with emotional eating (B = 0.0407, *p* < 0.001), whereas anxiety symptoms were not significantly associated with emotional eating (B = 0.0074, *p* = 0.499). The total effect of emotional stability on emotional eating was significant (B = −0.0261, *p* < 0.001), whereas the direct effect was not significant after accounting for anxiety and depressive symptoms included in the path model (B = −0.0101, *p* = 0.058). Bootstrap analyses indicated a significant total indirect effect (B = −0.0160). Examination of the specific indirect effects showed that the indirect effect through anxiety alone was not significant (B = −0.0021), whereas the indirect effect through depression alone (B = −0.0074) and the serial indirect effect through anxiety followed by depression (B = −0.0065) were both significant.

**Figure 3:**
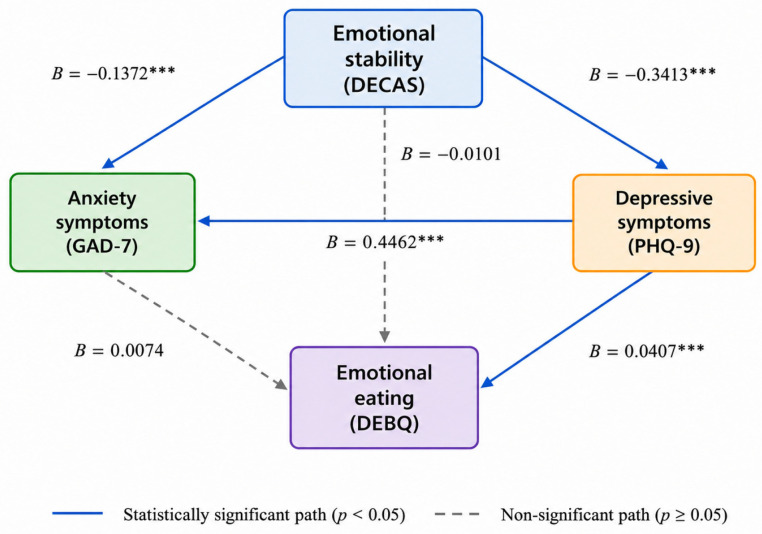
The serial path model with depressive symptoms specified before anxiety symptoms (PROCESS Model 6). The values on the arrows represent unstandardized regression coefficients (B). The solid arrows indicate statistically significant paths (*p* < 0.05), whereas the dashed arrows indicate non-significant paths. DECAS = DECAS Personality Inventory; PHQ-9 = Patient Health Questionnaire-9; GAD-7 = Generalized Anxiety Disorder-7; DEBQ = Dutch Eating Behavior Questionnaire. *** *p* < 0.001.

The results of the serial path analysis with depressive symptoms specified before anxiety symptoms are presented in Table 8 and Table 9, Figure 3. Emotional stability was significantly associated with lower levels of depressive symptoms (B = −0.3413, *p* < 0.001) and anxiety (B = −0.1372, *p* < 0.001), while depressive symptoms were significantly associated with anxiety (B = 0.4462, *p* < 0.001). Depressive symptoms were significantly associated with emotional eating (B = 0.0407, *p* < 0.001), whereas anxiety symptoms were not significantly associated with emotional eating (B = 0.0074, *p* = 0.499). The total effect of emotional stability on emotional eating was significant (B = −0.0261, *p* < 0.001), whereas the direct effect was not significant after accounting for anxiety and depressive symptoms included in the path model (B = −0.0101, *p* = 0.058). Bootstrap analyses indicated a significant total indirect effect (B = −0.0160). Examination of the specific indirect effects showed that only the indirect effect of emotional stability on emotional eating through depressive symptoms was statistically significant (B = −0.0139), whereas the indirect effect through anxiety alone (B = −0.0010) and the serial indirect effect through depressive symptoms and anxiety (B = −0.0011) were not statistically significant.

## 5. Discussion

This study explored whether anxiety and depressive symptoms explain the association between emotional stability and emotional eating among Romanian medical students. The results indicate that depressive symptoms showed the strongest statistically indirect statistical association in the path analyses. Although anxiety symptoms were associated with emotional eating at the correlational level, its independent indirect effect disappeared after depressive symptoms were taken into account. Interestingly, only the serial path model in which anxiety symptoms were specified before depressive symptoms showed a significant indirect effect, suggesting that depressive symptoms may be more closely associated with emotional eating within the specified statistical model.

Overall, these findings suggest that depressive symptoms showed the strongest indirect statistical association between emotional stability and emotional eating among medical students.

Emotional stability was negatively correlated with higher levels of anxiety and depressive symptoms in our sample. This finding is consistent with previous studies showing that emotional stability is an important predictor of the development of anxiety and depressive symptoms [47]. Studies conducted among medical students have shown that constant exposure to specific stressors, such as high academic demands, frequent assessments, a competitive environment, and early clinical exposure, can amplify psychological vulnerability, especially in students with low levels of emotional stability, favoring the development of anxiety and depressive symptoms [48,49,50]. Individuals with lower emotional stability experience negative emotions more intensely, have greater difficulty adapting to stressful situations, and are more vulnerable to developing anxiety and depressive symptoms [51]. In addition, individuals with lower emotional stability interpret events as more threatening than they are, use less effective emotional regulation strategies, and experience greater rumination [18,52].

Anxiety and depressive symptoms were positively correlated with emotional eating in our sample. There are numerous studies in the literature that support the fact that increased levels of anxiety and depression are associated with emotional eating [53]. Similarly, the review conducted by Alexatou et al. among university students found that emotional eating is associated with more severe symptoms of anxiety and depression [54]. One possible explanation is that individuals with anxiety or depressive symptoms may use emotional eating as a coping mechanism by consuming highly palatable foods to temporarily diminish negative affect and psychological distress [55,56]. Although this behavior may provide short-term relief by activating the brain reward system, it does not address the underlying source of distress and may ultimately reinforce a vicious cycle in which negative emotions promote emotional eating [12].

Emotional stability was negatively correlated with higher levels of emotional eating. These results are consistent with previous studies, which show that people with reduced emotional stability are more likely to use food as a strategy to regulate negative emotions and temporarily reduce psychological distress [57]. Individuals with lower emotional stability generally experience greater emotional reactivity and less adaptive coping strategies, making emotional eating a more likely response to psychological distress [58,59].

Given these correlations, we further examined the indirect statistical associations involving anxiety and depressive symptoms using regression-based path analyses.

In the parallel path model, although emotional stability significantly predicted both anxious and depressive symptoms, only depressive symptoms showed a significant indirect association linking emotional stability and emotional eating. Although few studies have simultaneously investigated the indirect statistical associations involving anxiety and depressive symptoms in this relationship, our findings are consistent with previous studies employing regression-based path analyses, suggesting that the association between emotional stability and eating behavior may involve indirect statistical associations rather than being solely direct [60]. A possible explanation is that depressive symptoms reflect a more persistent state of negative affect and difficulties in emotional regulation, which may favor the use of emotional eating as a coping strategy [61,62]. In contrast, anxiety is associated with more heterogeneous behavioral responses to stress [63]. While some people increase their food intake during times of anxiety, others experience a decrease in appetite, which may reduce the overall effect of anxiety on emotional eating [64].

The results of the serial path analyses showed that only the model in which anxiety symptoms mediator were specified before depressive symptoms mediator was statistically significant. Although the cross-sectional design of the study does not allow causal or temporal inferences, previous longitudinal studies have suggested that anxiety symptoms may precede the development of depressive symptoms, although the relationship is currently considered bidirectional [45,46]. In this context, the serial path model was statistically consistent with the possibility that the association between anxiety and emotional eating may be exerted indirectly through depressive symptoms rather than through a direct pathway. In contrast, the absence of a significant indirect effect in the alternative serial path model suggests that depressive symptoms may represent the strongest indirect association linking emotional stability and emotional eating in our sample.

Although the present study focused on psychological variables, the findings may also be relevant from a nutritional perspective because emotional eating has been consistently associated with poorer diet quality, increased consumption of energy-dense foods, excess energy intake, and overweight or obesity [65]. Nevertheless, emotional eating is a multifactorial behavior, and the associations observed in the present study are likely influenced by additional psychological processes beyond anxiety and depressive symptoms. Factors such as perceived stress, maladaptive coping strategies, psychological inflexibility, and broader emotion regulation capacities may also contribute to these relationships and warrant investigation in future longitudinal studies [66,67,68].

## 6. Limitations

The present study presents several limitations that must be considered when interpreting the results. First, the cross-sectional design does not allow for establishing causal relationships among the investigated variables nor for confirming the temporal sequence assumed by the proposed path models. Longitudinal studies are needed to verify whether the identified relationships reflect real temporal mechanisms.

Second, all variables were assessed through self-report questionnaires, which may introduce errors associated with subjective reporting. Although the DECAS Personality Inventory includes internal validation scales that help identify inconsistent responses, the other instruments used do not include such control mechanisms.

In addition, participants were recruited from a single university center, and the predominance of female participants reflected the demographic composition of the medical student population at the study institution. Nevertheless, caution is warranted when extrapolating the findings to medical students from other universities or to populations with a substantially different sex distribution. Future multicenter studies including students from several medical schools are needed to determine whether the observed associations are consistent across different academic settings and more diverse student populations.

Furthermore, because participation was voluntary and recruitment was conducted through student social media groups, the possibility of self-selection bias cannot be excluded.

Finally, dietary intake, diet quality, and metabolic outcomes were not assessed. Future studies may benefit from integrating these nutritional variables alongside psychological assessments.

## 7. Conclusions

The results of the present study indicate that emotional stability is an important factor associated with anxiety and depressive symptoms, as well as emotional eating, among medical students. The regression-based path analyses suggested that depressive symptoms showed the strongest indirect statistical association linking emotional stability and emotional eating. Within the proposed statistical models, anxiety was indirectly associated with emotional eating through depressive symptoms. These findings suggest that anxiety and depressive symptoms may represent important psychological factors associated with emotional eating among medical students. These findings may be useful in informing strategies to promote psychological well-being among medical students, including accessible mental health support, routine screening, and stress-management initiatives.

## Figures and Tables

**Table 1:** The characteristics of the study sample.

Characteristic	n = 291
Sex, n (%)	
Female	225 (77.32)
Male	66 (22.68)
Year of study, n (%)	
First year	37 (12.71)
Second year	21 (7.22)
Third year	23 (7.90)
Fourth year	27 (9.28)
Fifth year	52 (17.87)
Sixth year	131 (45.02)
Place of residence, n (%)	
Urban	219 (75.26)
Rural	72 (24.74)
Living arrangement, n (%)	
Dormitory	74 (25.43)
Rented accommodation	145 (49.83)
Living with family	46 (15.81)
Living alone	26 (8.93)
Age, years	
Mean ± SD	23.34 ± 2.06

Notes: SD = standard deviation; n = total number of study participants.

**Table 2:** The descriptive statistics of the study variables.

Variable	Mean ± SD	Median (IQR)
ES	47.88 ± 9.94	49 (12)
Anxiety symptoms	11.10 ± 5.36	11 (9)
Depressive symptoms	11.03 ± 6.03	11 (10)
EE	2.41 ± 0.87	2.31 (1.23)

Notes: ES = emotional stability; EE = emotional eating; SD = standard deviation; IQR = interquartile range.

**Table 3:** The Spearman correlation matrix of the study variables.

Variable	ES	Anxiety Symptoms	Depressive Symptoms	EE
ES	-			
Anxiety symptoms	−0.546 ***	-		
Depressive symptoms	−0.603 ***	0.668 ***	-	
EE	−0.383 ***	0.380 ***	0.454 ***	-

Notes: ES = emotional stability; EE = emotional eating; *** = *p* < 0.001. Correlations were calculated using Spearman’s rank correlation coefficient (ρ).

**Table 4:** The regression coefficients for the adjusted parallel path model (PROCESS Model 4).

Pathway	B	R^2^	SE	t	*p*	95% CI
ES → depressive symptoms	−0.3413	0.3773	0.0287	−11.906	<0.001	−0.3978, −0.2849
ES → anxiety symptoms	−0.2895	0.3650	0.0257	−11.246	<0.001	−0.3402, −0.2388
Depressive symptoms → EE	0.0407	0.3828	0.0098	4.141	<0.001	0.0214, 0.0601
Anxiety symptoms → EE	0.0074		0.0110	0.677	0.499	−0.0141, 0.0290
ES → EE (direct effect)	−0.0101		0.0053	−1.907	0.058	−0.0204, 0.0003
ES → EE (total effect)	−0.0261	0.3238	0.0043	−6.060	<0.001	−0.0346, −0.0176

Notes: ES = emotional stability; EE = emotional eating; B = unstandardized regression coefficient; SE = standard error; t = t statistic; CI = 95% confidence interval. Analyses were adjusted for sex, age, year of study and BMI.

**Table 5:** The indirect effects for the adjusted parallel path model (PROCESS Model 4).

Indirect Pathway	B	Boot SE	Boot LLCI	Boot ULCI
**Total indirect effect**	**−0.0160**	**0.0040**	**−0.0244**	**−0.0085**
ES → anxiety symptoms → EE	−0.0021	0.0032	−0.0086	0.0043
**ES → depressive symptoms → EE**	**−0.0139**	**0.0039**	**−0.0223**	**−0.0068**

Notes: B = unstandardized indirect effect; Boot SE = bootstrap standard error; Boot LLCI = lower limit of the bias-corrected 95% bootstrap confidence interval; Boot ULCI = upper limit of the bias-corrected 95% bootstrap confidence interval. Bootstrap estimates were based on 5000 resamples. Analyses were adjusted for sex, age, year of study and BMI. Bold values indicate statistically significant indirect effects (95% bootstrap confidence interval does not include zero).

**Table 6:** The regression coefficients for the adjusted serial mediation path model with anxiety specified before depressive symptoms (PROCESS Model 6).

Pathway	B	R^2^	SE	t	*p*	95% CI
ES → anxiety symptoms	−0.2895	0.3650	0.0257	−11.246	<0.001	−0.3402, −0.2388
ES → depressive symptoms	−0.1811	0.5311	0.0299	−6.048	<0.001	−0.2400, −0.1222
Anxiety → symptoms → depressive symptoms	0.5534		0.0573	9.651	<0.001	0.4406, 0.6663
Depressive symptoms → EE	0.0407	0.3828	0.0098	4.141	<0.001	0.0214, 0.0601
Anxiety symptoms → EE	0.0074		0.0110	0.677	0.499	−0.0141, 0.0290
ES → EE (direct effect)	−0.0101		0.0053	−1.907	0.058	−0.0204, 0.0003
ES → EE (total effect)	−0.0261	0.3238	0.0043	−6.060	<0.001	−0.0346, −0.0176

Notes: ES = emotional stability; EE = emotional eating; B = unstandardized regression coefficient; SE = standard error; t = t statistic; CI = 95% confidence interval. Analyses were adjusted for sex, age, year of study and BMI.

**Table 7:** The indirect effects for the adjusted serial path model with anxiety specified before depressive symptoms (PROCESS Model 6).

Indirect Pathway	B	Boot SE	Boot LLCI	Boot ULCI
**Total indirect effect**	**−0.0160**	**0.0040**	**−0.0243**	**−0.0087**
ES → anxiety symptoms→ EE	−0.0021	0.0033	−0.0087	0.0041
**ES → depressive symptoms → EE**	**−0.0074**	**0.0023**	**−0.0127**	**−0.0034**
**ES → anxiety → symptoms → depressive symptoms → EE**	**−0.0065**	**0.0019**	**−0.0109**	**−0.0032**

Notes: B = unstandardized indirect effect; Boot SE = bootstrap standard error; Boot LLCI = lower limit of the bias-corrected 95% bootstrap confidence interval; Boot ULCI = upper limit of the bias-corrected 95% bootstrap confidence interval. Bootstrap estimates were based on 5000 resamples. ES = emotional stability; EE = emotional eating. Analyses were adjusted for sex, age, year of study and BMI. Bold values indicate statistically significant indirect effects (95% bootstrap confidence interval does not include zero).

**Table 8:** The regression coefficients for the serial path with depressive symptoms specified before anxiety symptoms (PROCESS Model 6).

Pathway	B	R^2^	SE	t	*p*	95% CI
ES → depressive symptoms	−0.3413	0.3773	0.0287	−11.906	<0.001	−0.3978, −0.2849
ES → anxiety symptoms	−0.1372	0.5218	0.0274	−5.010	<0.001	−0.1911, −0.0833
Depressive symptoms → anxiety symptoms	0.4462		0.0462	9.651	<0.001	0.3552, 0.5372
Depressive symptoms → EE	0.0407	0.3828	0.0098	4.141	<0.001	0.0214, 0.0601
Anxiety symptoms→ EE	0.0074		0.0110	0.677	0.499	−0.0141, 0.0290
ES → EE (direct effect)	−0.0101		0.0053	−1.907	0.058	−0.0204, 0.0003
ES → EE (total effect)	−0.0261	0.3238	0.0043	−6.060	<0.001	−0.0346, −0.0176

Notes: ES = emotional stability; EE = emotional eating; B = unstandardized regression coefficient; SE = standard error; t = t statistic; CI = 95% confidence interval. Analyses were adjusted for sex, age, year of study and BMI.

**Table 9:** The indirect effects for the adjusted serial path model with depressive symptoms specified before anxiety symptoms (PROCESS Model 6).

Indirect Pathway	B	Boot SE	Boot LLCI	Boot ULCI
**Total indirect effect**	**−0.0160**	**0.0039**	**−0.0238**	**−0.0086**
**ES → depressive symptoms → EE**	**−0.0139**	**0.0039**	**−0.0221**	**−0.0067**
ES → anxiety symptoms→ EE	−0.0010	0.0016	−0.0044	0.0019
ES → depressive symptoms → anxiety symptoms → EE	−0.0011	0.0017	−0.0046	0.0022

Notes: B = unstandardized indirect effect; Boot SE = bootstrap standard error; Boot LLCI = lower limit of the bias-corrected 95% bootstrap confidence interval; Boot ULCI = upper limit of the bias-corrected 95% bootstrap confidence interval. Bootstrap estimates were based on 5000 resamples. ES = emotional stability; EE = emotional eating. Analyses were adjusted for sex, age, year of study and BMI. Bold values indicate statistically significant indirect effects (95% bootstrap confidence interval does not include zero).

## Data Availability

The data presented in this study are not readily available due to ethical and privacy considerations. Requests to access the datasets should be directed to the corresponding author.

## Abbreviations

The following abbreviations are used in this manuscript:

B	Unstandardized regression coefficient
BMI	Body mass index
Boot LLCI	Lower limit of the bias-corrected 95% bootstrap confidence interval
Boot SE	Bootstrap standard error
Boot ULCI	Upper limit of the bias-corrected 95% bootstrap confidence interval
CI	Confidence interval
DECAS	DECAS Personality Inventory
DEBQ	Dutch Eating Behavior Questionnaire
EE	Emotional eating
ES	Emotional stability
GAD-7	Generalized Anxiety Disorder-7
IQR	Interquartile range
PHQ-9	Patient Health Questionnaire-9
PROCESS	PROCESS macro for SPSS
SD	Standard deviation
SE	Standard error
SPSS	Statistical Package for the Social Sciences
VIF	Variance inflation factor
